# The complete chloroplast genome of the salt-tolerant plant *Kalidium caspicum* (Amaranthaceae) and evolutionary relationships

**DOI:** 10.1080/23802359.2026.2742623

**Published:** 2026-10-05

**Authors:** Xianhao Chen, Su Wang, Yanbing Chen, Wei Liang, Xiaodong Ding

**Affiliations:** Key Laboratory of Ecological Protection of Agro-Pastoral Ecotones in the Yellow River Basin, National Ethnic Affairs Commission of the People’s Republic of China, School of Biological Science and Engineering, North Minzu University, Yinchuan, China

**Keywords:** Salt-tolerant plants, *Kalidium caspicum*, chloroplast genome, phylogenetic analysis

## Abstract

*Kalidium caspicum* belongs to the genus *Kalidium* (Amaranthaceae). We assembled and annotated its chloroplast genome for the first time. The chloroplast genome exhibits a circular quadripartite structure, with a total length of 153,693 bp and a GC content of 36.3%. It contains 130 genes, including 85 protein-coding genes, 37 tRNA genes, and eight rRNA genes. Phylogenetic analysis consistently recovered *K. caspicum*, *K. foliatum*, and *K. gracile*, *Salicornia bigelovii*, and *S. fruticosa* as a well‑supported clade. This study clarifies the chloroplast genome characteristics and phylogenetic position of *K. caspicum*, providing a basis for molecular identification and phylogenetic research in *Kalidium*.

## 1. Introduction

*Kalidium caspicum* (Linnaeus) Ungern-Sternberg (Linnaeus 1753) is a semi-shrub belonging to the genus *Kalidium* within the the tribe Salicornieae of the subfamily Salicornioideae within the family Amaranthaceae (sensu lato) (Zhu 1995; Liu et al. 2022). This species is mainly distributed in northern Xinjiang, China, where it thrives in saline-alkali soils, desert margins, and salt lake shores (Wang et al. 2015). Its fleshy assimilating branches confer high tolerance (Yin et al. 2002), and it improves soil organic matter, thereby serving as a dominant species for the biological control of salinization and an advantageous species in salt-tolerant desert communities (Qu et al. 2008). As a succulent forage plant, *K. caspicum* forms large clusters, grows vigorously, and achieves the highest hay yield in autumn. In addition, the seeds can be ground into powder for human consumption or livestock feed (Wang et al. 2015). Despite *K. caspicum* plays a critical role in ecological and economic, the chloroplast genome of K. caspicum has not been reported, and its evolutionary relationships and genetic diversity remain unexplored.

Chloroplasts are essential plant organelles for photosynthesis and carbon fixation (Qin et al. 2023; Li et al. 2025). The chloroplast genome conserved structure and uniparental inheritance, the chloroplast genome has become a valuable tool in comparative genomics, phylogenetics, and species identification (Clegg et al. 1994; Nock et al. 2019; Quan et al. 2024). It is increasingly used as a super barcode to assess genetic and nucleotide diversity, resolve complex phylogenetic, and infer evolutionary history, including in Amaranthaceae family (Lauterbach et al. 2019; Cheng et al. 2021; She et al. 2022).

In this study, we assembled the complete chloroplast genome of *K. caspicum* for the first time. Our primary aims were to (1) characterize the sequence characteristics of the chloroplast genome of *K. caspicum* and its differences in chloroplast genome features compared with closely related species; and (2) the phylogenetic relationships among *Kalidium* species and the systematic position of *K. caspicum*.

## 2. Material and methods

Leaves of *K. caspicum* were collected from the Midong District (Urumqi, Xinjiang, China; coordinates: 87.6562° E, 44.1629° N), dried in silica gel, and used for DNA extraction (Figure 1A, 1B). Voucher specimens were identified by Dr. Lei Zhang (zhangsanshi-0319@163.com) and deposited in the Herbarium of North Minzu University (https://www.cvh.ac.cn/ins/info.php?code=NMU) under accession number zlnmu2025117 (Figure 1C). Total genomic DNA was isolated using the cetyltrimethylammonium bromide (Arseneau et al. 2017). Whole-genome survey sequencing data generated on an Illumina NovaSeq 6000 platform (San Diego, CA, USA), generating at least 6 Gb of data. Sequencing depth was assessed using SAMtools (Li et al. 2009).

**Figure 1.**
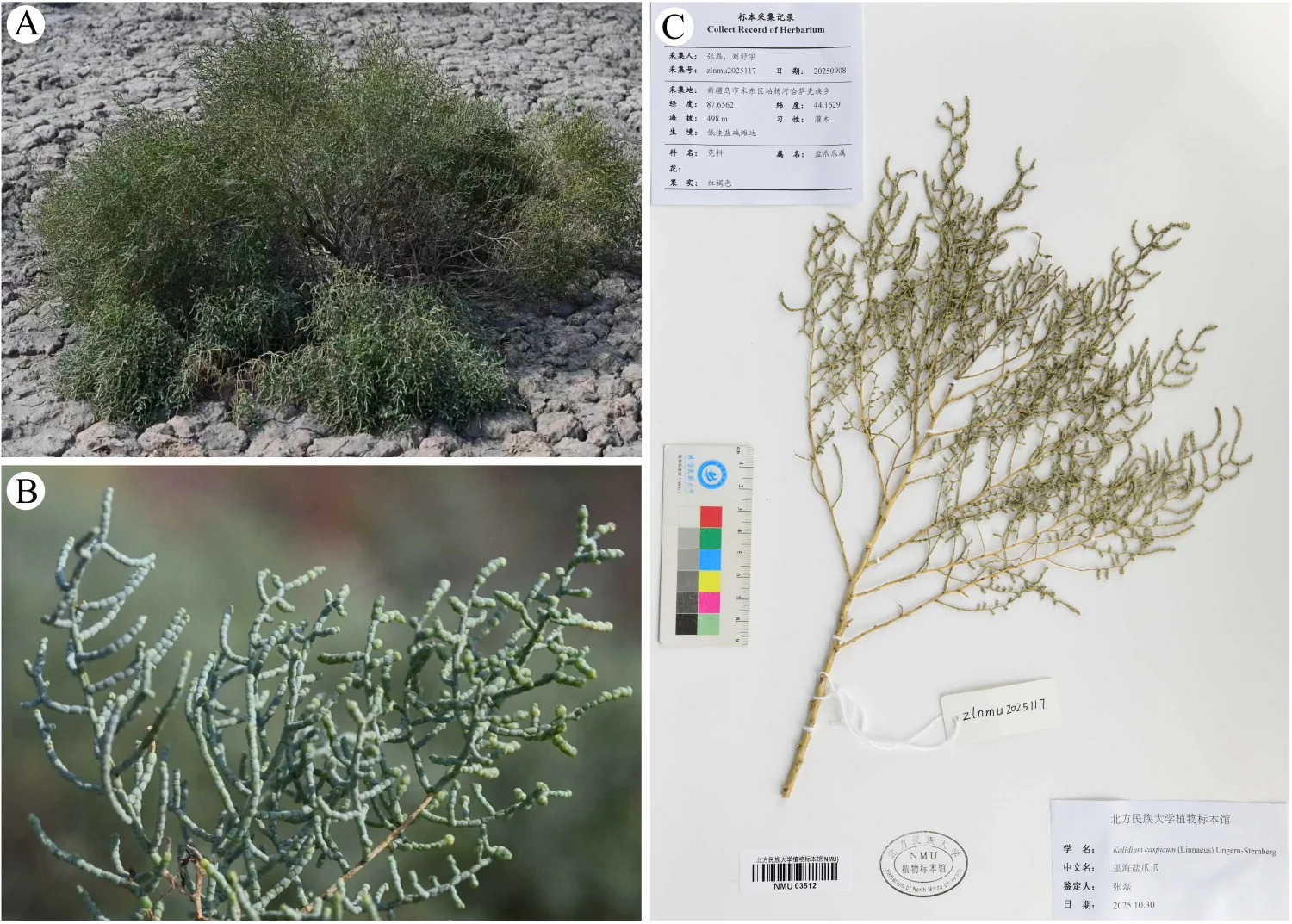
Photos of *K. caspicum*. (A) Field photos of *K. caspicum* (photographed by Dr. Xianhao Chen). (B) fleshy assimilating branches and leaves of *K. caspicum.* (C) Herbarium of *T. K. caspicum.*

The average quality score < 7 or containing > 10% ambiguous nucleotides reads were removed, and the remaining high-quality data were assembled using NOVOPlasty v2.7.2 (Dierckxsens et al. 2017): k-mer 39, seed from *K. gracile*, genome range 100-200kb, insert size 300 bp, and read length 150 bp. The assembly was further optimized by mapping the cleaned reads back to the the reference genome of *K. gracile* (ON149858) using Geneious v9.1.8 (Kearse et al. 2012) with default parameters. The complete chloroplast genome was annotated with Plann v1.1 (Huang and Cronk 2015) using the same reference, and then manually corrected with Sequin v15.10 software (Lehwark and Greiner 2018) to ensure accurate start/stop codons and exon-intron boundaries. A circular map was generated using OGDRAW v1.2 (Lohse et al. 2013). The final annotated sequence was submitted to GenBank (Sayers et al. 2020).

Protein-coding genes (PCGs) were extracted from the 20 chloroplast genomes. PCGs were aligned with PRANK v130410 (Löytynoja and Goldman 2008) based on amino acid translations (default parameters: gap opening 0.025, extension = 0.75, 5 iterations). Alignments were manually checked and concatenated into a supermatrix. Maximum likelihood (ML) in RAxML v8.1.24 (Stamatakis 2014) under the GTR + Γ with 1,000 bootstrap replicates; Bayesian inference (BI) in MrBayes v3.2.6 (Ronquist et al. 2012). employing the GTR+I + G model selected by jModeltest, with two independent MCMC runs of 5,000,000 generations, sampling every 500 generations. Trees were visualized using FigTree v1.4.2 (Rambaut 2012).

RSCU values were computed using CodonW (Li et al. 2025; Wu et al. 2025). High‑frequency codons (RSCU > 1) were identified, and for each gene, the one with the highest RSCU and frequency was chosen as its optimal codon (Wu et al. 2025). The results were visualized with the RSCU-Plot tool (https://pcg-lab.shinyapps.io/RSCU-Plot/) (Akrami et al. 2025; Diani and Soorni 2025; Hejazi et al. 2025; Soorni and Golchini 2025). Simple sequence repeats (SSRs) were characterized using MISA v2.1 (https://webblast.ipk-gatersleben.de/misa/) (Beier et al. 2017) with parameters of ≥ 10 repeats for mononucleotides, ≥ 5 for dinucleotides, ≥ 4 for trinucleotides, and ≥ 3 for tetra-, penta-, and hexanucleotides. Results were visualized in R v4.3.2 (Zhang et al. 2013) with the Circos package v0.69-9 (Villanueva and Chen 2019). The junction sites of the four structural regions (IRA, LSC, SSC, and IRB) and adjacent genes were compared across three *Kalidium* PCGs using IRscope (Amiryousefi et al. 2018) to obtain a macroscopic view of the chloroplast genome structure.

## 3. Results

Following quality control and preprocessing, at least six gigabases (Gb) of high‑quality clean whole-genome sequencing data were assembled into the complete chloroplast genome of *K. caspicum* (PZ511203) using a reference-guided assembly approach. The assembled genome is 153,693 bp in length (Figure 2), with maximum, minimum, and average sequencing depths of 997 ×, 23 ×, and 730.89 ×, respectively (Figure S1). It exhibited the typical quadripartite structure, comprising a large single-copy (LSC) region of 84,899 bp, a small single-copy (SSC) region of 18,854 bp, and two inverted repeats (IRa and IRb) of 24,970 bp (Figure 2). The *K. caspicum* chloroplast genome encodes 130 predicted genes, include 85 protein coding genes, 37 tRNA genes, and eight rRNA genes. Among these genes, nine protein-coding genes (*rpl*2, *rps*7, and *ycf*2), six tRNA genes (*trn*I-GAU, *trn*L-CAA, *trn*V-GAC, *trn*R-ACG, and *trn*N-GUU), and four rRNA genes (*rrn*4.5, *rrn*5, *rrn*16, and *rrn*23) were duplicated in the IR regions (Table S1). Additionally, the chloroplast genome contained 11 cis-splicing genes (Figure S2) and 1 trans-splicing gene (Figure S3). The GC content of the *K. caspicum* chloroplast genome was determined to be 36.3% overall. with values of 34.1% in the LSC, 29.5% in the SSC, and 42.8% in the IR regions.

**Figure 2.**
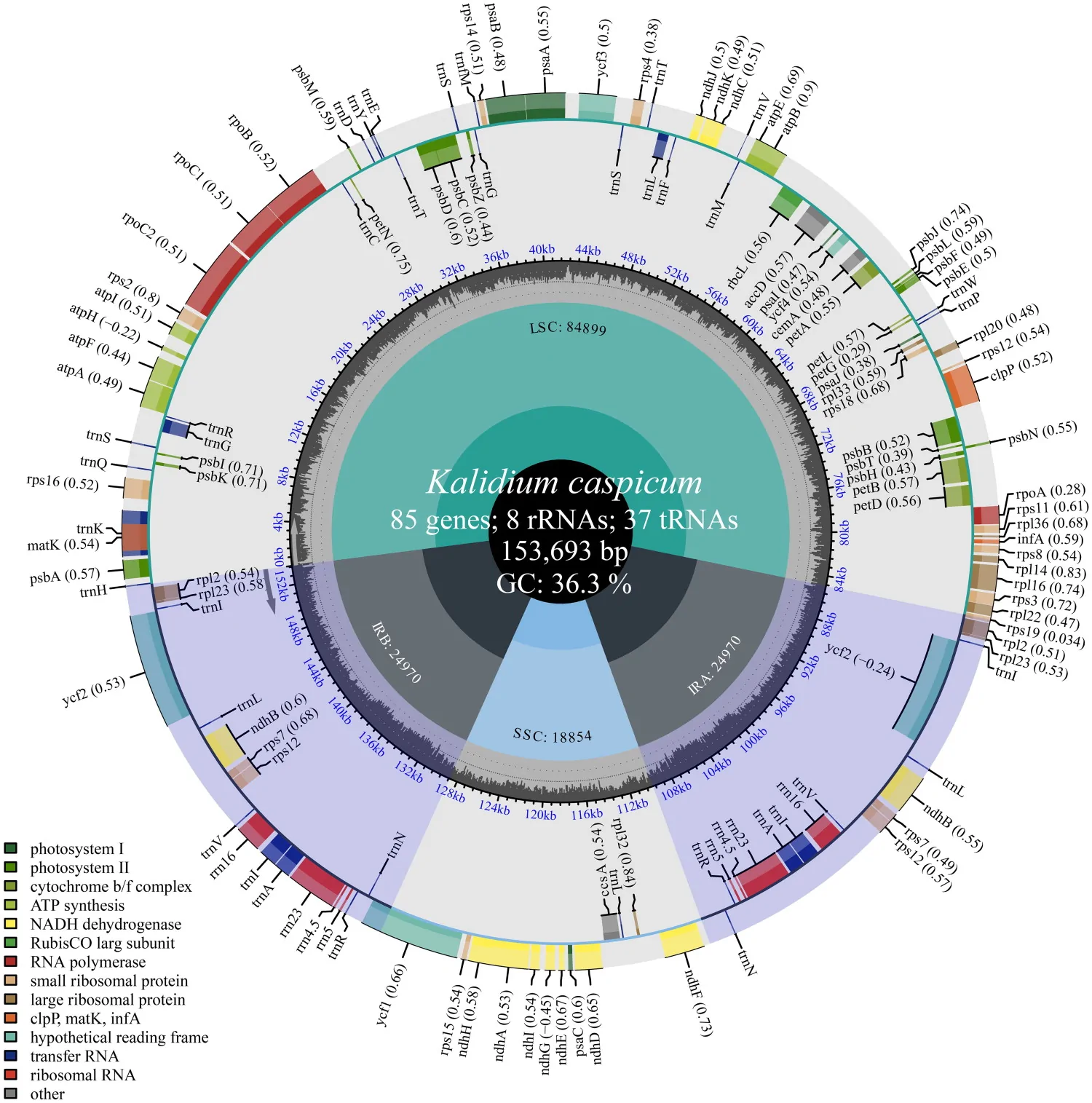
Circular map of the *K. caspicum* chloroplast genome. The outer track uses colored boxes to represent protein-coding, tRNA, and rRNA genes. Bold lines on the inner circle delineate the inverted repeat regions (IRA and IRB), which separate the genome into the small single-copy (SSC) and large single-copy (LSC) regions. Genes shown on the outer and inner sides of the outer circle are transcribed clockwise and counterclockwise, respectively. The innermost track displays the GC content (light gray).

We analyzed the complete chloroplast genomes of 20 Amaranthaceae species, including 19 Spirolobeae and one Cyclolobeae, The final concatenated dataset, after trimming poorly aligned regions and gaps resulting from missing genes, comprised 71 PCGs and 56,686 sites (Table S2). Maximum Likelihood (ML) and Bayesian inference (BI). The inferred phylogenies exhibited congruent topologies with strong nodal support, dividing the Spirolobeae species into four major branches. Among them, *K. caspicum*, *K. foliatum*, and *K. gracile*, along with their sister species *Salicornia bigelovii* and *S. fruticosa* consistently recovering a well‑supported clade (Figure 3). This robust phylogenetic reconstruction firmly establishes the taxonomic placement of *K. caspicum* within the genus *Kalidium* (Amaranthaceae).

**Figure 3.**
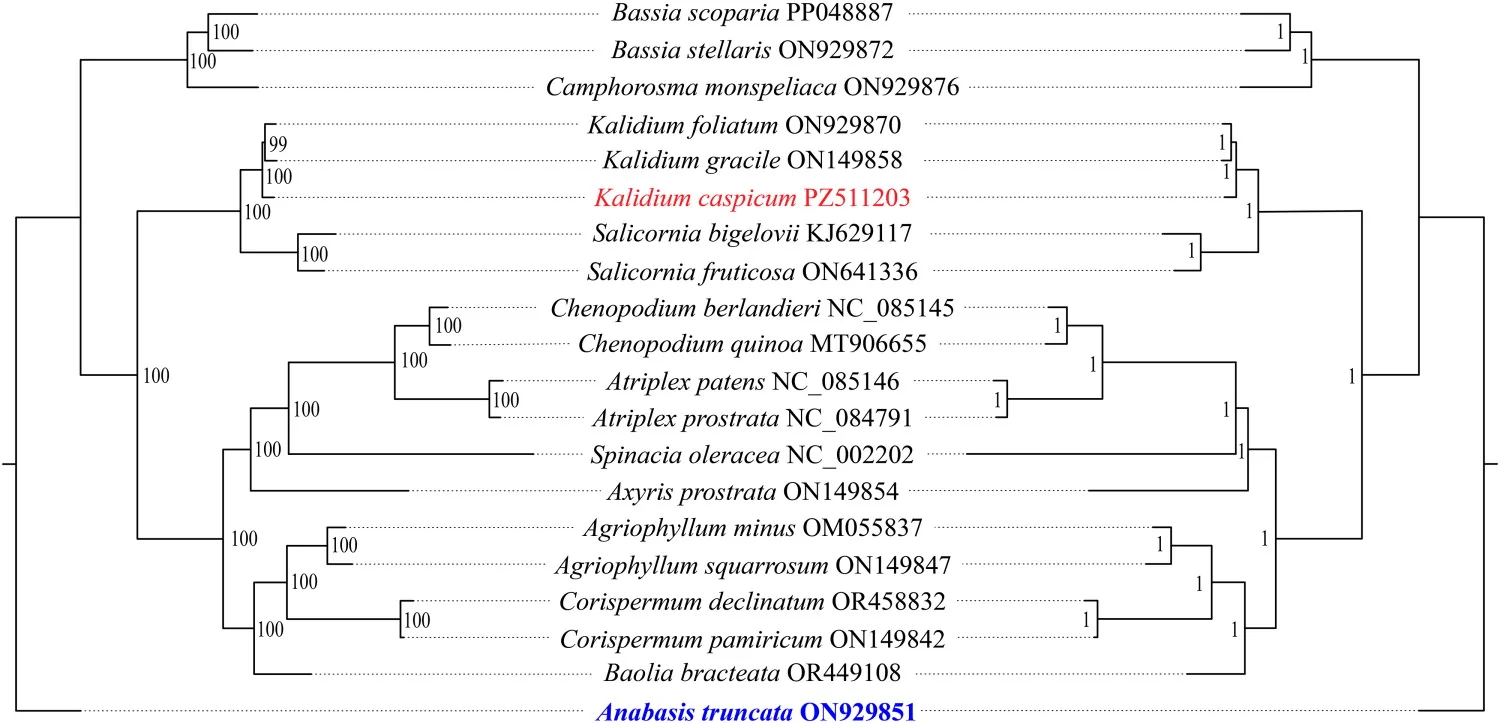
Phylogenetic tree based on 71 PCGs for spirolobeae species with *anabasis truncata* as the outgroup (bold and blue). The left and right trees represent the results of maximum likelihood (ML) and bayesian inference (BI) analyses, respectively. Branch support values are shown as ML bootstrap percentages (e.g. 100) on the left tree and as BI posterior probabilities (e.g. 1.0) on the right tree. GenBank accession numbers: *Bassia scoparia* PP048887 (Wan et al. 2024), *bassia stellaris* ON929872 (Wan et al. 2024), *camphorosma monspeliaca* ON929876 (Liu et al. 2026), *kalidium foliatum* MW699755 (Wang et al. 2022), *kalidium gracile* ON149858 (Osmonali et al. 2023), *kalidium caspicum* (PZ511203), *salicornia bigelovii* KJ629117 (Jamdade et al. 2022), *salicornia fruticosa* ON641336 (Samigullin et al. 2025), *chenopodium berlandieri* NC_085145 (Wei et al. 2023), *chenopodium quinoa* MT906655 (Ren et al. 2022), *atriplex patens* NC_085146 (Wei et al. 2023), *atriplex prostrata* NC_084791 (Wei et al. 2023), *spinacia oleracea* NC_002202 (Schmitz-Linneweber et al. 2001), *axyris prostrata* ON149854 (Sukhorukov et al. 2022), *agriophyllum minus* OM055837 (Genievskaya et al. 2017), *agriophyllum squarrosum* ON149847 (Genievskaya et al. 2017), *corispermum declinatum* OR458832 (Liu et al. 2024). The species from this study are shown in red on the phylogenetic tree.

## 4. Discussion and conclusion

This study reports the first complete chloroplast genome of the *K. caspicum*. The assembled genome is 153,693 bp in length and contains 130 genes, falling within the typical angiosperm plastomes (Qu et al. 2019; Yao et al. 2019; Alshegaihi 2024). Notably, chloroplast genome of *K. caspicum* are highly similar to other *Kalidium* species (Figure S4), reinforcing a conserved structural framework. The lack of major IR rearrangements suggests that chloroplast genome is stable within the genus.

Phylogenetic reconstruction robustly places *K. caspicum*, *K. foliatum*, and *K. gracile* in a well‑supported clade together with their sister taxa *Salicornia bigelovii* and *S. fruticosa* (Kadereit et al. 2012; Brignone et al. 2022; Liu et al. 2026). This topology implies that either *Kalidium* as currently circumscribed is paraphyletic, or *Salicornia* represents a derived lineage within a broader *Kalidium* alliance.

RSCU analysis revealed that the majority of which exhibited a pronounced preference for A/U at the third codon position (Figure S5). This pattern is consistent with the generally AT‑rich nature of cp genomes and may reflect mutational pressure rather than translational selection. The SSRs identified here provide valuable molecular markers for future population genetics and conservation studies (Figure S6). Overall, our work enriches the genomic resources for *Kalidium* and offers baseline data for evolutionary and ecological research.

## Supplementary Material

Supplementary File.docx

## Data Availability

The *K. caspicum* chloroplast genome sequence data that support the findings of this study are openly available in NCBI (https://www.ncbi.nlm.nih.gov/) under the accession no. PZ511203. The associated BioProject, SRA and Bio-Sample numbers are PRJNA1471537, SRR38923952 and SAMN60455879, respectively.
