# Supplementary material for: The complete chloroplast genome of the salt-tolerant plant Kalidium caspicum (Amaranthaceae) and evolutionary relationships
Source: Mitochondrial DNA B Resour. 2026 Oct 5;11(11):1266–71. doi: 10.1080/23802359.2026.2742623 (PMC13644432; doi:10.1080/23802359.2026.2742623)
Supplement: Supplementary file 1 [file TMDN_A_2742623_SM0336.docx]

**Table S1** Functional classification of chloroplast genes of *Kalidium caspicum*

|  | Gene family | Gene name |
| --- | --- | --- |
| Photosynthesis related genes | Subunits of photosystem I(5) | *psa*A, B, C, I, J |
|  | Subunits of photosystem II(15) | *psb*A, B, C, D, E, F, H, I, J, K, L, M, N, T, Z |
|  | Subunits of ATP synthase(6) | *atp*A, B, E, F*, H, I |
|  | Subunits of NADH-dehydrogenase(12) | *ndh*A*, B*(×2), C, D, E, F, G, H, I, J, K |
|  | Subunits of cytochrome b/f complex(6) | *pet*A, B*, D*, G, L, N |
|  | Subunit of rubisco(1) | *rbc*L |
|  | Subunit of Acetyl-CoA-carboxylase(1) | *acc*D |
| Transcription and translationrelated genes | Large subunit of ribosome(11) | *rpl*14, 16, 2(×2), 20, 22, 23(×2), 32, 33, 36 |
|  | Small subunit of ribosome(14) | *rps*11, *12***(×2), 14, 15, 16*, 18, 19, 2, 3, 4, 7(×2), 8 |
|  | DNA dependent RNA polymerase(4) | *rpo*A, B, C1*, C2 |
|  | RNA Ribosomal RNAs(8) | *rrn*4.5 (×2), 5 (×2), 16 (×2), 23 (×2) |
|  | RNA Transfer RNAs(37) | *trn*H-GUG, K-UUU*, Q-UUG, S-GCU, G-UCC*, R-UCU, C-GCA, D-GUC, Y-GUA, E-UUC, T-GGU, S-UGA, G-GCC, M-CAU, S-GGA, T-UGU, L-UAA*, F-GAA, V-UAC, M-CAU, W-CCA, P-UGG, I-CAU(×2), L-CAA(×2), V-GAC(×2), I-GAU*(×2) , A-UGC*(×2), R-ACG (×2), N-GUU (×2), L-UAG |
| Others genes | c-type cytochrom synthesis gene(1) | *ccs*A |
|  | Envelop membrane protein(1) | *cem*A |
|  | Maturase(1) | *mat*K |
|  | Protease(1) | *clp*P** |
|  | Translational initiation factor(1) | *infA* |
| Unknown function genes | Conserved open reading frames(5) | *ycf*1, 2 (×2), 3**, 4 |

#* represents a gene with one intron,** represents a gene with two introns,×2 indicates two copies.

**Table S2 Comparison of protein-coding genes**

| **gene** | **Total site num** | **Pi site num** | **Percent of Pi site (%)** |
| --- | --- | --- | --- |
| atpA | 1521 | 160 | 10.519 |
| atpE | 399 | 49 | 12.281 |
| atpF | 573 | 89 | 15.532 |
| atpH | 243 | 14 | 5.761 |
| atpI | 741 | 56 | 7.557 |
| clpP | 588 | 184 | 31.293 |
| matK | 1590 | 523 | 32.893 |
| ndhA | 1095 | 160 | 14.612 |
| ndhB | 1560 | 56 | 3.59 |
| ndhC | 360 | 40 | 11.111 |
| ndhD | 1527 | 292 | 19.122 |
| ndhE | 303 | 37 | 12.211 |
| ndhF | 2592 | 922 | 35.571 |
| ndhG | 528 | 69 | 13.068 |
| ndhH | 1182 | 162 | 13.706 |
| ndhI | 552 | 122 | 22.101 |
| ndhJ | 492 | 62 | 12.602 |
| petA | 966 | 106 | 10.973 |
| petB | 657 | 71 | 10.807 |
| *pet*D | 480 | 39 | 8.125 |
| petG | 111 | 6 | 5.405 |
| petL | 93 | 10 | 10.753 |
| petN | 87 | 6 | 6.897 |
| psaA | 2250 | 151 | 6.711 |
| psaC | 243 | 24 | 9.877 |
| psbA | 1059 | 76 | 7.177 |
| psbB | 1524 | 148 | 9.711 |
| psbC | 1419 | 99 | 6.977 |
| psbD | 1059 | 65 | 6.138 |
| psbE | 249 | 16 | 6.426 |
| psbF | 117 | 6 | 5.128 |
| psbH | 243 | 49 | 20.165 |
| psbI | 168 | 67 | 39.881 |
| psbJ | 120 | 7 | 5.833 |
| psbK | 201 | 32 | 15.92 |
| psbL | 114 | 4 | 3.509 |
| psbM | 150 | 55 | 36.667 |
| psbN | 129 | 6 | 4.651 |
| psbT | 114 | 22 | 19.298 |
| rbcL | 1479 | 208 | 14.064 |
| rpl14 | 366 | 38 | 10.383 |
| rpl16 | 408 | 72 | 17.647 |
| rpl2 | 945 | 553 | 58.519 |
| rpl20 | 387 | 72 | 18.605 |
| rpl23 | 315 | 55 | 17.46 |
| rpl32 | 180 | 60 | 33.333 |
| rpl33 | 198 | 31 | 15.657 |
| rpl36 | 111 | 11 | 9.91 |
| rpoB | 3213 | 344 | 10.707 |
| rpoC1 | 2088 | 289 | 13.841 |
| rpoC2 | 4245 | 920 | 21.673 |
| rps11 | 414 | 55 | 13.285 |
| rps14 | 300 | 28 | 9.333 |
| rps15 | 270 | 51 | 18.889 |
| rps16 | 273 | 53 | 19.414 |
| rps18 | 378 | 205 | 54.233 |
| rps2 | 840 | 229 | 27.262 |
| rps3 | 672 | 128 | 19.048 |
| rps4 | 603 | 62 | 10.282 |
| rps7 | 465 | 14 | 3.011 |
| rps8 | 411 | 65 | 15.815 |
| ycf2 | 7296 | 3213 | 44.038 |
| ycf3 | 504 | 26 | 5.159 |
| ycf4 | 588 | 157 | 26.701 |

**Figure S1** Coverage depth distribution of the *K. caspicum*.

**Figure S2** Structure of Cis-splicing genes in the *K. caspicum*.

**Figure S3** Structure of trans-splicing genes in the *K. caspicum*.

**Figure S4** Comparisons of the borders of the large single-copy (LSC), small single-copy (SSC), and inverted repeat (IR) regions among the CPGs of three *Kalidium* species.

**Figure S5** Heatmap of codon clustering for *K. caspicum* and close relatives species.

**Figure S6** Distribution of SSRs in five species.
